# The trend and features of physician workforce supply in China: after national medical licensing system reform

**DOI:** 10.1186/s12960-018-0278-8

**Published:** 2018-04-03

**Authors:** Chengxiang Tang, Daisheng Tang

**Affiliations:** 1 School of Public Administration, Guangzhou University, Guangzhou, 510320 Guangdong China; 2 China Center for Health Economic Research, National School of Development, Peking University, Beijing, 100871 China; 3 School of Economics and Management, Beijing Jiaotong University, Haidian, Beijing, 100044 China

**Keywords:** Physician supply, Licensing, Medical examination, China

## Abstract

**Background:**

The annual number of newly licensed physicians is an important indicator of medical workforce supply, which can accurately reflect an inflow into the health care market over a period. In order to both regulate medical professions and improve the quality of health care services, China established its medical licensing system from the point of the implementation of ‘Law on Practising Doctors’ in 1999. The objective of this study is to depict the trend and structure of newly licensed physicians thereafter.

**Methods:**

This study analyses a unique census data set that provides the headcount of newly licensed physicians from 2005 to 2015 in China. We also review a short history of medical licensing system reform in China since the 1990s.

**Results:**

The annual number of first-time licensed physicians in China increased from 159 489 in 2005 to 221 639 in 2015. Up to 2015, over half of newly licensed physicians had not received a medical education equivalent to a bachelor degree or higher. Around 51% of China’s newly licensed physicians were female in 2005, while the same ratio for females in 2015 was 56%.

**Conclusion:**

This article first provides an exploratory analysis of physician inflow into health care market in China using physician licensing data. The medical licensing system in China allows entering physicians with a broad range of educational levels. Moreover, the feminisation of the physician supply in China has become increasingly apparent and its impacts on health care provision still require more rigorous examination.

## Introduction

The health care industry is a labour-intensive sector, and thus, human resources for health planning is a critical process in developing strategies to meet health care demands [1]. Among the various types of human resources for health, the physician workforce is an essential part [2]. Therefore, monitoring changes of the trend and characteristics of the physician supply deserves more attention by both policymakers and researchers, because the current size of supply flow is critical in setting policies to address a shortage or oversupply of physicians. Specifically, this is rather important in China to examine these changes in physicians because the country has a huge ageing population that implies increasing health care demands need to be met [3].

A potentially less difficult way to the above monitoring of supply approach is to investigate the annual number of newly licensed physicians, which is formulated by the medical occupational regulation [4]. Through the lens, we can precisely observe the inflow of first-time licensed physicians into the health workforce over a certain time period. Many countries have developed a similar way to monitor the supply, attrition and retention of the physician workforce [5–7]. However, there has been no such study charting of the physician workforce supply in terms of newly licensed physicians over time since the launch of the medical licensing system in China in 2001.

This study aims to address this gap in the literature. To explore the trend and features of physicians, we first reviewed a short history of medical licensing system reform in China since the 1990s. Our study further analyses a unique census data set that provides the headcount of newly licensed physicians from 2005 to 2015 in China. Specifically, this study aims to provide an aggregate description of the trend and current situation in the field of the physician workforce supply in China. This paper offers a useful snapshot of the recent supply of the physician workforce flow, suggesting trends over time that may benefit many different stakeholders in China.

## Background

### History and origin of the licensing system

Like many other countries, occupational regulation in China generally consists of two approaches that include certification and licensing [8]. To put it simply, the certification system allows any person to practise the relevant job and uncertified individuals carry no legal consequences for practising. The licensing system, however, is a type of more restrictive occupational regulation, in which performing a job without a license is illegal and will be punished. In China, both certification and licensing for the health workforce are regulated by the governments. Moreover, an individual entering into a licensing occupation often requires basic educational or training prerequisites, qualifications in licensing examinations, or even a couple of years of internship and residency in professional facilities.

The modern occupational regulation in China was initiated in the 1950s, and at that time, the certification of occupations prevailed. There was no functioning labour market under the planned economy in China before the 1980s, in which rationed entry of medical students generally made the licensing system irrelevant for the physician workforce. The licensing system in occupational regulation was first introduced in China by the labour department of central government in the 1990s [9–11]. Unlike China, the licensing regulation system, including licensing examinations, has been implemented in the UK and other major European countries for over a century. Even compared with some east-Asian countries and regions, China has a much shorter history in medical licensing—Japan and Korea started such occupational regulation licensing system in the 1940s; Taiwan and Hong Kong adopted similar regulations thereafter [12, 13].

The common justification in economic theory for occupational regulation, especially the licensing of medical professions, is to guarantee a minimum quality of standards [14, 15]. Due to information asymmetry prevailing in medical practice, it is difficult for consumers to assess the basic level of health care quality, whereas it is an alternative—and easier—option to regulate standards of human resources input in health care production. Theoretically and practically, a medical licensing system aims to ensure that doctors have required knowledge and skills to practise medicine for patients safely and effectively [16].

### China’s medical licensing reform

There were only a few occupations, such as accounting, architecture and medicine that implemented licensing regulations in the middle of the 1990s. Because of its importance for the population’s health and its prestige in society, the occupation of physicians was one of the first to introduce licensing regulations. The Doctors Qualification Examination in China was first introduced in 1996 to evaluate and certify the applicant’s professional knowledge. The 9th National People’s Congress approved the ‘Law on Practising Doctors’ in 1998 and brought it into force on May 1, 1999. However, the complete medical examination that tests the skills of clinical practice was not implemented 2 years later in 2001.

This reform around 2000 legitimately established the medical licensing system for China and addressed the call for improving the quality of a trained workforce after a school-based medical education in the 1990s [17]. At present, a doctor in China who wants to get a full license to practise medicine should meet a minimum number of requirements, including graduation from an accredited medical school, residency experience or the successful completion of a period of practice under supervision of licensed doctors, and a National Medical Examination qualification certificate [18]. However, residency or internship training programmes that attempt to cover all medical graduates in China is still under development [19].

Due to the adoption of a national medical licensing system, it should be easier to illustrate the trend and features of the physician supply. The number of people who obtained licensing certificates consistently predicts the supply of the physician workforce [6], regardless of whether doctors are defined as those who are qualified in a licensing examination and are registered at a county or higher-level health authority as either licensed doctors or licensed assistant doctors.

Previously, the enrolment of medical students could not precisely predict the physician supply when a licensing system was absent. In a well-known study published by *The Lancet*, Anand and his colleagues estimated that nearly one third (206 000 out of 674 000) of college and university graduates in medical-related fields during 2000–2005 were not practising as doctors [3]. Therefore, the annual number of newly licensed physicians is an important indicator of medical workforce supply, which can accurately reflect an inflow into the health care market over a certain period of time.

## Methods

### Data sources

In view of the scope and objectives of this study, it was important to collect information from a reliable source. Instead of collecting information from reports and yearbooks published by the government [20], the National Medical Examination Centre of China maintains a comprehensive, central repository of data from every province involved with the applicants. The database contains gender, age, educational and specialty category information, as well as examination qualification information about every candidate who sits exam. The repository is unique in that it is the only national database consisting of the most up-to-date information from every province that has granted new physicians a medical license to practice in the authority’s region. In order to obtain an accurate count of new physicians, our study gathers the census data set that provides the number of newly licensed physicians from 2005 to 2015.

### Descriptive analyses

The main outcome measure was the number of newly licensed doctors in broad occupational groups, including clinical physicians (both licensed physician and licensed assistant physician), dentists (both licensed physician and licensed assistant physician) and public health physicians (both licensed physician and licensed assistant physician) overall.^1^ The newly licensed doctors are those who have passed both the written examination and the clinical skills test. In China, an individual becomes a licensed physician generally after the successful completion of the National Medical Examination and following registration at the practising site. In our study, however, we do not differentiate the number of physicians who obtained new licenses and the number of physicians who registered locally to practise after becoming licensed. One more point needs to be noted: that is, that the data did not include the category of physicians who practise Traditional Chinese Medicine. As discussed in the introduction, the number of newly licensed physicians is a consistent and most important indicator to illustrate the supply flow of the physician workforce, as it is believed that an individual needs to invest a large amount of time and effort to pass the National Medical Examination and thus to obtain a physician’s license [6, 7].

This study used simple headcounts of newly licensed physicians to calculate and chart the trends from 2005 to 2015. In the original data set, there are eight categories of educational level. We deleted three categories, namely traditional medicine or traditionally specialised expertise, no record of formal medical schooling, and that of data not available (less than 0.01% in total). Then we merged the remaining five categories into three in the final calculation, which were bachelor or above, vocational diploma level and second vocational diploma (SVD). The level of bachelor or above refers to at least 4 years’ training at formal medical universities. We aggregated bachelor, master degree and PhD levels because the latter two only account for around 3% for each year. The vocational diploma level indicates 3 years of medical education at junior colleges. One more important difference between bachelor level and vocational diploma level is the average quality of entering students, in which the average scores for admission into the former school courses are substantially higher than those of the latter courses, based on national college entrance examination (CEE).

One point which needs to be noted is that the International Standard Classification of Occupations (ISCO) and the WHO have defined and required that both General/Specialist Medical Practitioners require completion of a university-level degree (or a Bachelor) in basic medical education [21, 22]. However, this definition contradicts with the facts in China. Based on ‘The Law for Licensing Medical Practitioner’ (1999), the officials allow medical graduates from non-university, non-degree-oriented program to become assistant doctors, then assistant doctors have the chance to obtain the full doctor licensure after they accumulate certain years of work experience and pass an examination. It is expected that this explanation could be helpful to avoid any confusion.

The data also excluded three cases that reported no information on the gender in 2014. The percentages of new physicians by age, medical educational level and gender were also calculated. All statistics were descriptive. Stata 14 and Microsoft Excel 2010 were used for all analyses.

## Results

### Newly licensed physicians—age structure

The age profile of newly licensed physicians is illustrated in Table 1. There are five age groups for those candidates who passed the National Medical Examination. The largest age group is equal to or greater than 50 years old; the youngest age group is equal to or less than 20 years old. As charted in the figure, the proportion of physicians aged 20–30 accounts for the largest part of first-time licensed physicians. This proportion, however, declined from 76% in 2005 to less than 70% in 2014. The share of new physicians aged between 30 and 40 years is generally beyond 20%, while the shares are roughly consistent over the period of 2005 to 2015. The supply of new physicians aged 40–50 gradually increased from only 1% to nearly 6%. Moreover, both the oldest and youngest age groups have the least shares (less than 1%).

**Table 1:** Percentage change of age composition in new physicians from 2005 to 2015 (%)

Year	Age < 20	20<=Age < 30	30<=Age < 40	40<=Age < 50	50<=Age
2005	0.17	76.07	21.96	1.70	0.11
2006	0.08	75.54	22.41	1.86	0.10
2007	0.05	73.93	23.90	2.02	0.10
2008	0.03	73.46	24.18	2.25	0.08
2009	0.03	73.98	23.43	2.45	0.12
2010	0.02	71.12	25.34	3.41	0.12
2011	0.03	72.52	24.00	3.36	0.08
2012	0.03	70.85	24.83	4.14	0.15
2013	0.04	71.56	23.70	4.50	0.20
2014	0.03	69.65	24.66	5.43	0.24
2015	0.04	71.15	22.85	5.67	0.29

### Newly licensed physician—educational background

**Fig. 1:**
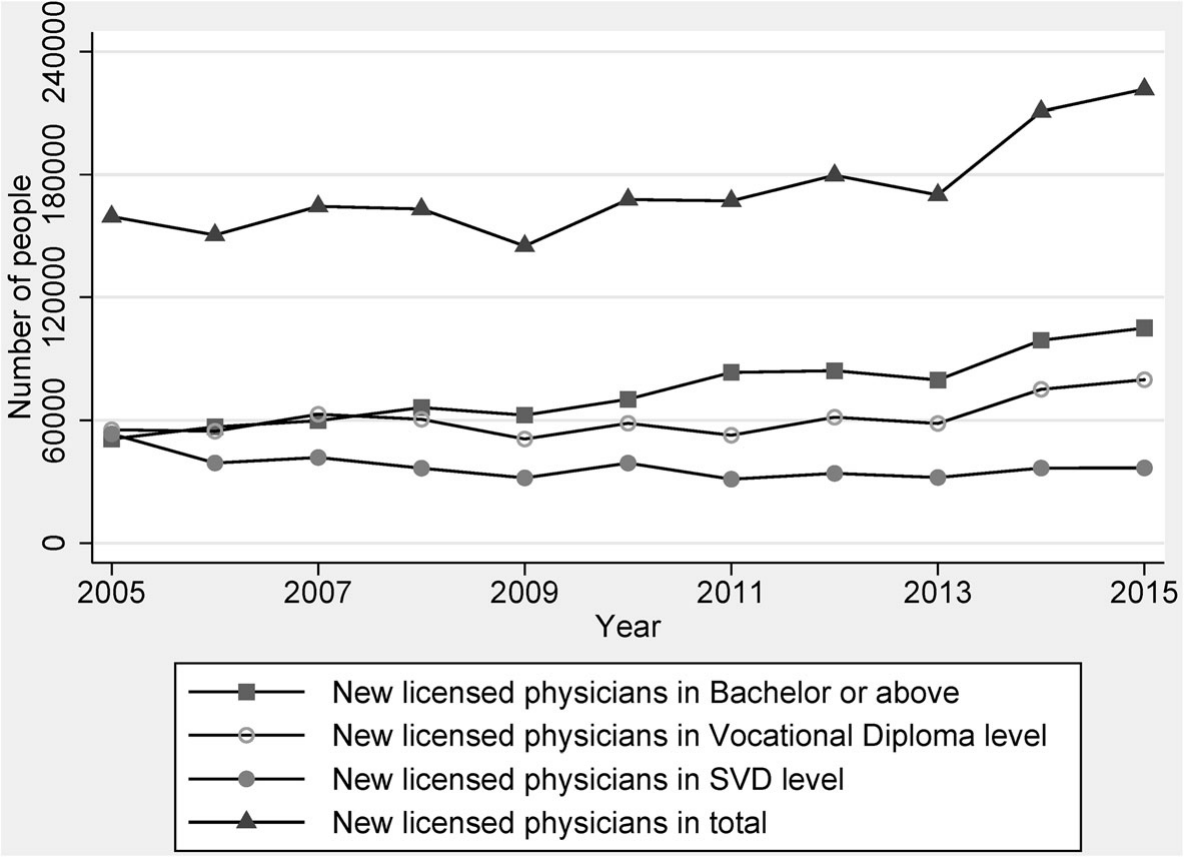
Time trend of number of new licensed physicians from 2005 to 2015. Note: There is a minor difference between the statistic number of new licensed physician and the final number of those physicians, because some provinces lower the national score for the examination in 2009

**Fig. 2:**
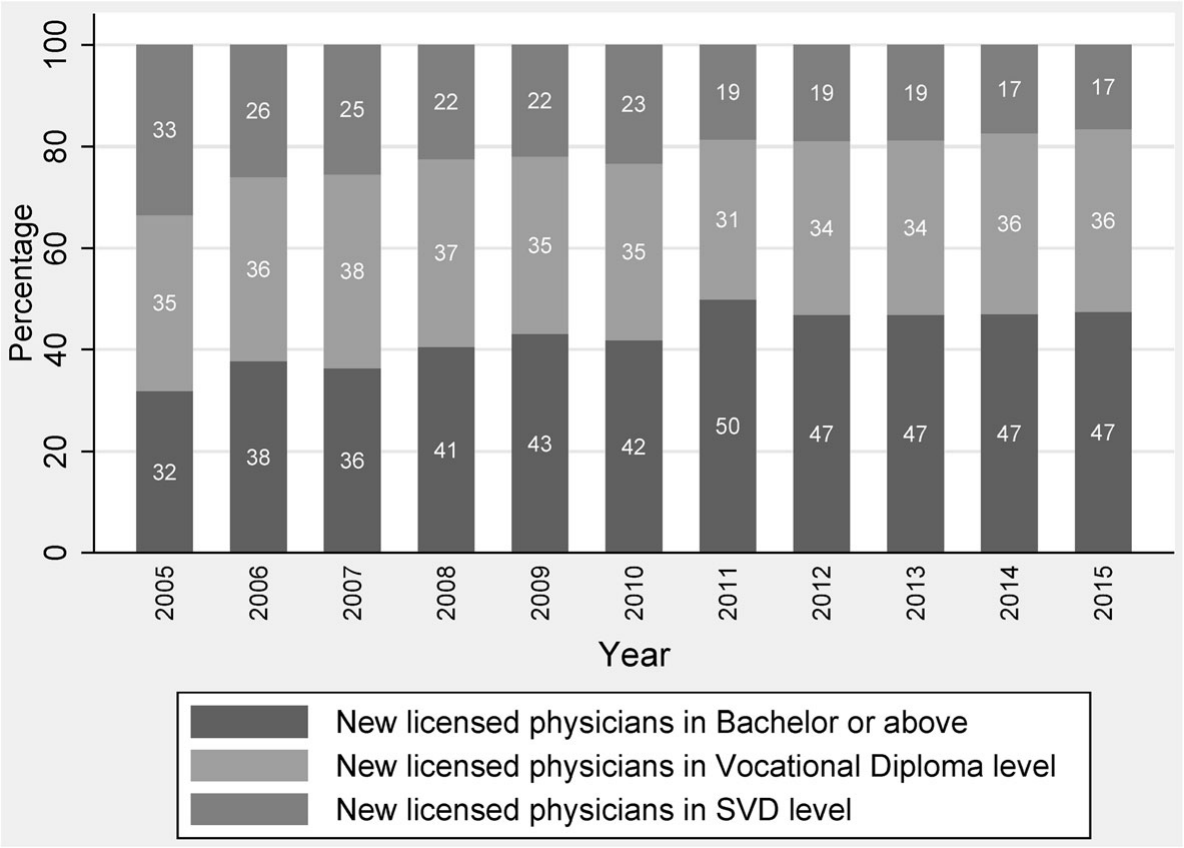
Percentage change of education composition in new physicians from 2005 to 2015

Figures 1 and 2 are presented for newly licensed physicians with their medical education level. Overall, the number of newly licensed physicians generally increased after the reform of the national medical licensing system. The annual number of first-time licensed physicians in China increased from 159 489 in 2005 to 221 639 in 2015. Among the three educational groups, the SVD or the least-educated group gradually decreased from 33% in 2005 to 17% in 2015.

Figure 2 shows that in 2005, one third of new physicians graduated from SVD medical schools, more than one third graduated from vocational diploma level medical schools, and nearly one third obtained a medical education equivalent to a bachelor degree or higher. Until 2015, the majority of new physicians (47%) had an entry-level or bachelor degree of medicine, whereas over half of newly licensed physicians had not received a medical education equivalent to a bachelor degree or the higher.

### Newly licensed physician—gender composition

**Fig. 3:**
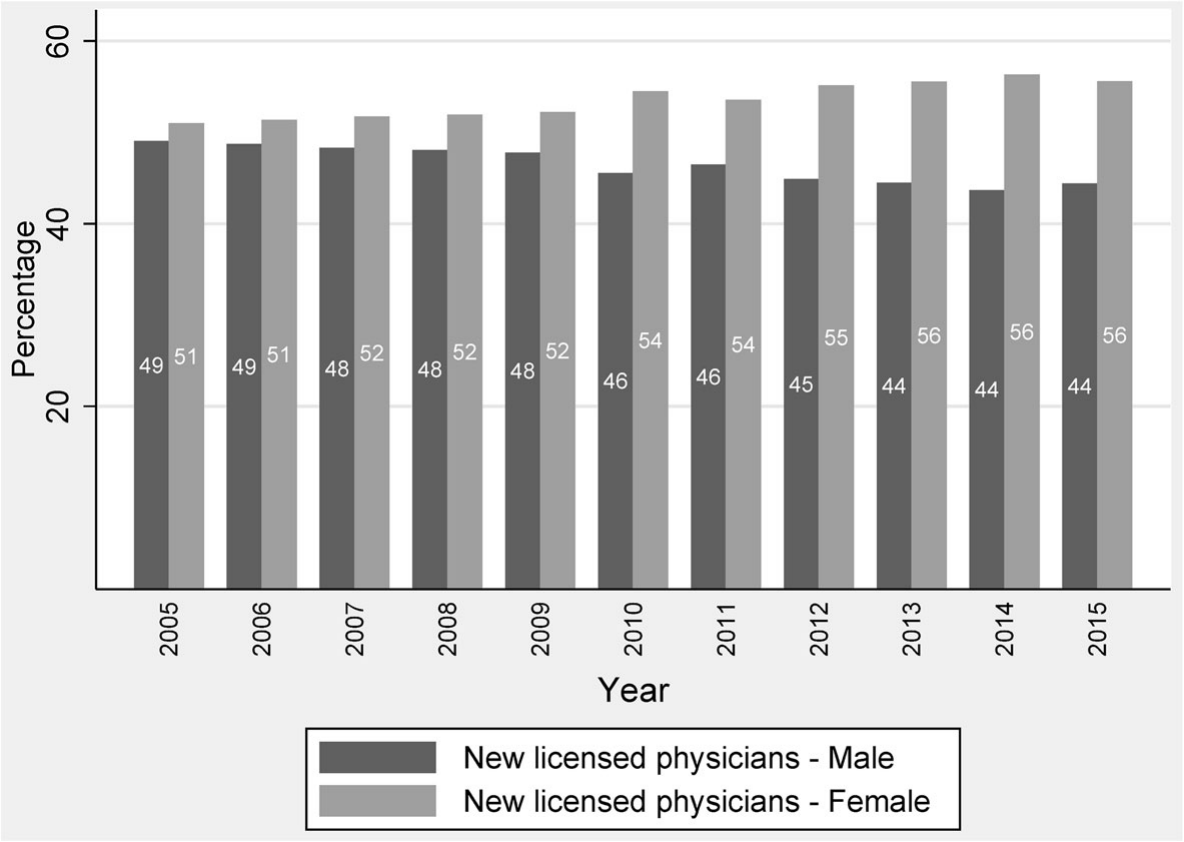
Percentage change of gender composition in new physicians from 2005 to 2015

Figure 3 illustrates the gender distribution of new physicians from 2005 to 2015. In 2005, around 51% of China’s newly licensed physicians were female, so at that time the percentage difference in gender was only 2%, whilst the same ratio for females in 2015 was 56%, showing a 12% difference between female and male physicians in the supply side. This trend in gender difference is gradually increasing, which may suggest a feminisation change in the physician workforce in China.

### Newly licensed physician—deployment in the facilities

Table 2 describes the deployment in various facilities of all newly licensed physicians. This table shows that the major employers of newly licensed physicians are hospitals and township hospitals. Before 2014, every licensed physician needed 1 year of work experience before taking the national medical examination, so every physician had been employed by a medical organization. From 2014, China launched a national residency programme, through which residents with 1 year of work experience were not employed by the facility. Therefore, a change in the employment of physicians resulted in 30.61 and 27.3% of newly licensed physicians who had no deployment information in 2014 and 2015, respectively.

**Table 2:** Newly licensed physician deployment in the facilities (%)

Facility type	2008	2009	2010	2011	2012	2013	2014	2015
Hospitals	56.13	55.11	50.86	55.02	53.38	54.26	39.04	42.07
Community health centres	3.38	4.12	3.88	4.07	4.32	4.32	3.33	3.18
Township hospitals	23.65	22.76	27.56	22.89	24.15	23.09	15.97	16.79
Clinics	9.17	10.88	10.74	10.79	11.22	11.92	8.26	7.75
Emergency departments	0.12	0.12	0.09	0.10	0.11	0.09	0.06	0.05
Blood banks	0.13	0.12	0.08	0.10	0.09	0.07	0.01	0.01
Maternal and children health care facilities	3.12	2.99	2.89	3.03	2.83	2.66	2.09	2.24
Specialised health institutes	0.58	0.60	0.52	0.49	0.46	0.39	0.26	0.24
CDCs	1.48	1.36	1.43	1.49	1.35	1.20	0.20	0.19
Health inspection institutes	0.00	0.00	0.00	0.00	0.00	0.00	0.00	0.00
Medial education facilities	0.00	0.00	0.00	0.00	0.00	0.00	0.00	0.00
Health promotion facilities	0.00	0.00	0.00	0.00	0.00	0.00	0.00	0.00
Other health facilities	2.23	1.95	1.96	2.02	2.11	2.00	0.16	0.17
NGO for health	0.00	0.00	0.00	0.00	0.00	0.00	0.01	0.01
Not available	0.00	0.00	0.00	0.00	0.00	0.00	30.61	27.30

## Discussion

In order to both regulate medical occupations and improve the quality of health care services, China established its medical licensing system with the implementation of the ‘Law on Practising Doctors’ in 2001. The present study aims to depict the trend and structure of newly licensed physicians thereafter. Our results show that the annual number of first-time licensed physicians in China increased from the year 2005 to 2015. In addition, over half of newly licensed physicians have not received a medical education equivalent to a bachelor degree until in 2015. Around 51% of China’s newly licensed physicians were female in 2005, while this ratio increased to 56% in 2015.

### Medical educational issue of newly licensed physicians

The results provide a number of new insights. The first topic that needs to be discussed is the heterogeneity of the medical education of entering physicians. Among those new physicians in 2015, half of them have not received a medical education equivalent to a bachelor degree. Nearly one out of five new physicians has completed only secondary level of education (SVD part). Over one out of three new physicians has received vocational diploma education at junior colleges. As mentioned above, both the quality of entering students to vocational diploma programs and the quality of the programs themselves are not a quarter as good as the quality of medical education equivalent to a bachelor degree.

Both theoretical and empirical studies have demonstrated that quality of physicians can significantly influence the quality of care. Theoretically, the milestone paper in health economics proposed that four variables in the market for physicians determine the health care provisions, which are price, the quality of entering medical students, the quality of medical education, and quantity [23]. A recent paper systematically reviewed relevant studies and also suggested that heterogeneous physicians impose several ‘hidden costs’ to the health sector in China, including transaction costs, trust, information costs, cost of defensive medicines, and additional time costs allocated to travel and long waiting time [24]. Empirically, a study using unannounced standardised patients found that poor-quality care in China’s rural clinics can be attributed to the educational attainment and medical qualification of clinicians [25].

This is uncommon phenomena in most high-income countries, as physicians are a homogeneous group in that they receive almost the same level of education training no matter where they practise. It is for certain that more professional health workers are needed in a developing country, like China, but it has become clear that efforts to scale up health professionals’ education should not only increase the quantity of doctors, but also ensure their quality in terms of medical education. A delicate balance between the quality of care (medical elitism) and access (equity of providers) exists, and China placed more weight on the latter [19]. A similar case is that the US Flexner Report in 1910 promoted the closure of medical schools at secondary level across all states [26]. As a result, our paper suggests that the reform of medical education is a priority for China to keep improving the performance of its health care system.

### Feminisation of the physician workforce supply

To the best of our knowledge, this is the first study that provides evidence on the feminisation of the physician workforce supply in China. If the trend of 12% difference between male and female new physicians keeps or even continues to increase, the inflow will definitely influence gender composition of the stock of physicians. This considerable phenomena of feminisation trend in the physician workforce has been explored in a number of studies in many developed and some developing countries, and it has been found that female physicians work fewer hours and with a lower workload than their male counterparts, especially in the primary care workforce [27–29]. A recent systematic review summarised the impact of the feminisation of the primary care physician workforce on service supply and showed a small negative impact of feminisation on the availability of primary health care services in developed countries [30]. Another empirical paper also investigated feminisation of the medical workforce in three African capital cities and found a trend of feminisation and anticipated more rigorous studies in low- and middle-income settings [31].

This paper can provide only a direct observation on the change of physicians; it is difficult to give any solid conclusion about its impact on health care provision in China utilising our data for at least two reasons: First, the degree of productive substitution that occurs between genders and the rate of feminisation still remain to be examined; second, the feminisation of various medical specialties should have more implications for a physician workforce productivity study. Thus, more studies on both the stock and dynamics of physicians’ gender composition are warranted in China.

### Next steps of reform

The National Medical Examination is an important progressive step in the implementation of the medical licensing system. At present, there are two major reforms of the examination that are under development. First, a pilot of a two-stage National Medical Examination that recruited 33 universities has been conducted. Previously, an applicant needed 1 year of work experience before he or she could take the one-stage exam. For medical students graduated from those 33 universities, there are separate stages of the exam before and after the 1-year residency programme [32]. Second, it has been reported that applicants should be granted two chances in the written examinations, given the low pass rate in the annual medical examination (around 20%) [33]. Nonetheless, it seems this policy may still be under discussion. In summary, reforms on improving both the quality and quantity of newly licensed physicians are in progress.

This is the first study to examine the supply trend and supply structure of newly licensed physicians after occupational regulation reform in China. Although this study is based on a national census data set that has hardly any bias, it was limited by only a few variables of newly licensed physicians. Due to a lack of data, it would be a great advantage if further topics could be explored in the near future, such as geographical distribution, specialty mix and mobility of newly licensed physicians. Moreover, the full-time equivalent of working hours is still a standard method of measuring productivity of health care and thus to describe the supply of health manpower and accurately project the physician workforce supply in China.

## Conclusion

This article provides an exploratory analysis of physician inflow into the health care market in China using the most recent physician licensing data. The establishment of a medical licensing system is undoubtedly an appropriate approach to control for the quantity of people allowed to enter the physician workforce with a minimum standard of education quality. Our investigation may inform policymakers of human resources for health in at least two aspects: first, policymakers need to pay more attention to the heterogeneity of the medical education of entering physicians; second, the feminisation of the physician supply in China has become increasingly apparent and its impacts on health care provision still require more rigorous examination. This study contributes a useful observation of changes in the physician workforce in China while being helpful in improving future policies on medical occupational regulation in terms of both quantity and educational structure.

## Footnotes

1 Licensing doctors includes four categories: clinical physicians, dentists, public health physicians and Traditional Chinese Medicine (TCM) doctors. This study did not include TCM physicians as they were categorized under CAM (Complementary and Alternative Medicine). Please refer to http://www.gov.cn/banshi/2005-08/02/content_19310.htm
